# Working memory capacity affects trade-off between quality and quantity only when stimulus exposure duration is sufficient: Evidence for the two-phase model

**DOI:** 10.1038/s41598-019-44998-3

**Published:** 2019-06-19

**Authors:** Chaoxiong Ye, Hong-Jin Sun, Qianru Xu, Tengfei Liang, Yin Zhang, Qiang Liu

**Affiliations:** 1 Institute of Brain and Psychological Science, Sichuan Normal University, 610000 Chengdu, China; 2 Department of Psychology, University of Jyvaskyla, 40014 Jyväskylä, Finland; 3 Department of Psychology, Neuroscience and Behaviour, McMaster University, L8S 4K1 Hamilton, Canada; 4 Research Center of Brain and Cognitive Neuroscience, Liaoning Normal University, 116029 Dalian, China

**Keywords:** Working memory, Human behaviour

## Abstract

The relation between visual working memory (VWM) capacity and attention has attracted much interest. In this study, we investigated the correlation between the participants’ VWM capacity and their ability to voluntarily trade off the precision and number of items remembered. The two-phase resource allocation model proposed by Ye *et al*. (2017) suggests that for a given set size, it takes a certain amount of consolidation time for an individual to control attention to adjust the VWM resources to trade off the precision and number. To verify whether trade-off ability varies across VWM capacity, we measured each individual’s VWM capacity and then conducted a colour recall task to examine their trade-off ability. By manipulating the task requirement, participants were instructed to memorise either more colours in a low-precision way or fewer colours in a high-precision way. We conducted two experiments by adjusting stimulus duration to be longer than predicted critical value (Experiment 1) and duration shorter than predicted critical value (Experiment 2). While the results of Experiment 1 showed a positive correlation between the VWM capacity and trade-off ability, the results of Experiment 2 showed a lack of such correlation. These results are consistent with the prediction from the two-phase model.

## Introduction

Visual working memory (VWM) is a system to actively maintain visual information and provide the information for advanced cognitive processing^1^. This system provides a storage space as a buffer to integrate information into continuous visual experience. By maintaining the mental contents after the visual scene disappears, this system plays a role as a gatekeeper between perception and high-level cognition^2^.

It is well known that VWM capacity develops in childhood, peaks in adulthood, and then declines with age^3,4^. Previous studies also found that VWM capacity differs substantially across different populations, and the VWM impairment is associated with a variety of cognitive disorders, such as Parkinson’s disorder^5^ and schizophrenia^6,7^. Moreover, there are reliable individual differences in VWM capacity even for healthy population^8^. Some individuals show VWM capacity of four items or above, whereas others show VWM capacity of two or less^9^. Besides, these individual differences are robustly correlated with many advanced cognitive functions such as fluid intelligence^10^. Therefore, understanding the relation between VWM capacity and other cognitive constructs is an essential topic in this field.

Many studies have investigated the relationship between individuals’ VWM capacity and attention control ability^11–16^. A well-established consensus is that individuals with low VWM capacity are poor at exerting attention control over what is being consolidated and maintained in VWM. For example, evidence provided by Vogel, *et al*.^13^ clearly illustrated this relationship. They identified a contralateral delay activity (CDA), an ERP component strongly modulated by the number of items in VWM during the maintenance phase and reaches an asymptote once VWM capacity is exhausted. They asked participants to memorise simple targets and ignore distractors. The CDA amplitude was used as an index of whether irrelevant distractors unnecessarily consumed VWM storage. The results suggested that participants with high VWM capacity were able to filter out the distractors, but low VWM capacity participants tended to fail to filter out distractors and involuntarily allocate VWM storage to them. By using a similar technique, a recent study also found that even when social stimuli such as emotional faces acted as distractors in a VWM task, high VWM capacity participants could still filter face distractors entirely, while participants with low VWM capacity automatically stored neutral and angry face distractors into VWM^14^. These studies have shown that low VWM capacity individuals could not help but orient their attention to distractors^11^, and thus they ended up storing more distractors in their limited-capacity VWM than high VWM capacity individuals^13^. Therefore, the individual’s VWM capacity is highly related to the attention efficiency for target selection (also see Cowan and Morey^17^). Although this individual difference approach successfully clarified the critical relationship between VWM capacity and attention control in the tasks with distractors^11,13,14,18^, it is still unclear how attention control ability is affected by the VWM capacity in other tasks.

In tasks without distractors, the attention control can be studied in tasks requiring a voluntary trade-off ability in VWM resource allocation. Here, the trade-off ability for VWM precision and number refers to a situation in which individuals adjust the VWM resource allocation voluntarily, while the lack of trade-off ability refers to a situation where individuals can only allocate VWM resource involuntarily. So far in the literature, there have been many studies on whether individuals could voluntarily trade off the VWM precision and number^19–28^, but the conclusion is still debated.

Zhang and Luck^23^ investigated whether participants could voluntarily trade off the VWM precision and number when they have enough incentives. In their Experiment 3, they asked participants to memorise four colours and then recall one of the colours. After the response in each trial, feedback would be presented to inform them about their performance. They gave participants different incentives to trade off the VWM precision and number by manipulating the rules of the feedback. In the low-precision condition, participants could get some rewards even when recalling colours with low-precision. On the contrary, in the high-precision condition, only when they reported the colour with high precision, they would be told that they were correct and received more monetary rewards. However, Zhang and Luck^23^ did not find any evidence to support the voluntary trade-off ability in individuals with their experimental setting. Similarly, Murray, *et al*.^19^ asked participants to memorise stimuli in a way that emphasised precision or number, and only observed the null effects. These results support that the resource could only be allocated in a stimulus-driven (involuntary) manner.

However, the phenomenon of voluntary trade-off has been reported in some other studies. In an ERP study, Gao, *et al*.^24^ asked participants to memorise orientations of items with either low-precision or high-precision. Their results showed that, in low-precision condition, participants could retain three to four items in VWM, but in high-precision condition, participants could only retain two items in VWM. Similarly, Machizawa, *et al*.^20^ found that participants could adjust the memory precision when the memory load was low (two items) according to different task requirements. These results suggest that people have a voluntary trade-off ability, and support that the resource could also be allocated in a goal-directed (voluntary) manner.

Recently, Ye, *et al*.^21^ proposed a two-phase VWM resource allocation model that can explain these two different patterns of results^19,20,23,24^. In their experiments, they used an orientation recall task. Similar to Experiment 3 and 4b in Zhang and Luck’s^23^ study, by manipulating the feedback following each trial, Ye, *et al*.^21^ asked participants to memorise different numbers of orientations in different precision blocks. The novel aspect of the study is that stimulus exposure duration and set size were systematically manipulated. They found no effect of precision requirement with a high set size and short exposure duration (stored four items with 200 ms exposure duration). However, when the set size was low (stored two items with 200 ms exposure duration) or the exposure duration was long (stored four items with 500 ms exposure duration), participants could trade off the VWM precision and number according to task requirements. These results were interpreted with the two-phase VWM resource allocation model. The model states that in the early phase the VWM resource could only be allocated in a stimulus-driven (involuntary) manner. However, the allocation could enter the late phase after the completion of the first phase. In the late phase, the allocation could be controlled flexibly according to task requirements in a goal-directed (voluntary) manner. In other words, individuals automatically allocate VWM resources in the early phase and create a low-precision representation for each item in the scope of attention. After the low-precision representations have been entirely created or the VWM resources have been completely allocated, the individual can reallocate VWM resources and create high-precision representations according to the requirements of the task. Therefore, for a given set size, it takes a certain amount of consolidation time for an individual to control attention to adjust resources to trade off the memory precision and number in the VWM task without distractors.

Table 1 summarises stimulus and result patterns found in ten published papers in the literature about the voluntary trade-off between VWM precision and number^19–28^. The table also reports whether these experiments support an individual’s voluntary trade-off for VWM precision and number. In 23 out of 25 experiments reported, when the exposure duration per stimulus item equalled or exceeded 100 ms/item, trade-off between the VWM number with precision occurred, however, when the exposure duration per stimulus item was equal or smaller than 50 ms/item, trade-off between the VWM precision with number did not occur. We thus speculate that the different patterns of results in terms of the trade-off between VWM precision and number could be determined by a critical value of the ratio of duration/set size (a value between 50–100 ms/item). The two-phase VWM resource allocation model could explain such critical value influencing the trade-off.

**Table 1:** A summary of the studies about the voluntary trade-off between VWM precision and number.

Study	Experiment	N	Task	Stimulus	Set size	Duration (ms)	Duration/set size (ms/item)	Trade off voluntarily?	Can the critical value hypothesis predict it?
Zhang and Luck^23^	Experiment 1a	13	Recall	Colour	4	200 ms	50 ms/item	No	Yes
	Experiment 1b	13	Recall	Colour	4	200 ms	50 ms/item	No	Yes
	Experiment 2	14	Recall	Colour	4	200 ms	50 ms/item	No	Yes
	Experiment 3	10	Recall	Colour	4	200 ms	50 ms/item	No	Yes
	Experiment 4	10	Recall	Colour	6	200 ms	33.3 ms/item	No	Yes
Gao *et al*.^24^		19	Change detection (CDA)	Orientation	2,4	500 ms	125, 250 ms/item	Yes	Yes
Murray *et al*.^19^	Experiment 1	12	Change detection	Orientation	4	200 ms	50 ms/item	No	Yes
	Experiment 2	20	Change detection	Orientation	4	200 ms	50 ms/item	No	Yes
	Experiment 3	20	Change detection	Orientation	4	200 ms	50 ms/item	No	Yes
	Experiment 4	20	Change detection	Orientation	4	200 ms	50 ms/item	No	Yes
Machizawa *et al*.^20^	Experiment 1	20	Change detection	Orientation	2 4	200 ms 200 ms	100 ms/item 50 ms/item	Yes No	Yes Yes
	Experiment 2	20	Change detection (CDA)	Orientation	2 4	200 ms 200 ms	100 ms/item 50 ms/item	Yes No	Yes Yes
Ye *et al*.^22^		14	Change detection (CDA)	Colour	2, 3, 4	100 ms	25–50 ms/item	No	Yes
He *et al*.^27^	Experiment 1	12	Change detection (CDA)	Colour	2, 4	200 ms	50–100 ms/item	No	Yes
	Experiment 2	21	Change detection (CDA)	Colour	2, 4	200 ms	50–100 ms/item	No	Yes
Fougnie *et al*.^26^	Experiment 1	18	Recall	Colour	5	1200 ms	240 ms/item	Yes	Yes
	Supplementary experiment	18	Recall	Colour	5	200 ms	40 ms/item	Yes	No
	Experiment 2	18	Recall	Colour	5	1200 ms	240 ms/item	Yes	Yes
Bocincova *et al*.^25^		60	Recall	Colour	2, 4	150 ms	37.5–75 ms/item	No	Yes
Ye *et al*.^21^	Experiment 1	47	Recall	Orientation	4	200 ms	50 ms/item	No	Yes
	Experiment 2	50	Recall	Orientation	2	200 ms	100 ms/item	Yes	Yes
	Experiment 3	47	Recall	Orientation	4	500 ms	125 ms/item	Yes	Yes
Ramaty and Luria^28^	Experiment 1	20	Recall	Colour	5	1200 ms	240 ms/item	Yes	Yes
	Experiment 2	20	Recall	Colour	5	300 ms	60 ms/item	No	Yes
	Experiment 3	20	Recall	Colour	5	1200 ms	240 ms/item	No	No

It is reasonable to argue that the critical value of duration/set size affecting trade-off could be related to individual participants’ VWM capacities. All ten studies described above examined the participants’ performance at a population level (using the mean performance of the sample). An alternative way to identify the critical factor affecting the trade-off is to examine the relation between trade-off tendency and *individual*’s capacity. Although so far in the literature there have been many studies examining whether individuals could trade off the VWM precision and number^19–28^, surprisingly, the impact of individual’s VWM capacity on the trade-off ability of VWM precision and number have not been carefully examined.

The current study takes a novel approach to examine the effect of individual VWM capacity on the underlying process in resource allocation. To be more comparable with Zhang and Luck^23^, we conducted a similar colour recall task to examine participants’ trade-off ability. In order to verify whether trade-off ability varies across VWM capacity, we first measured each individual’s VWM capacity and then conducted the colour recall task to examine their trade-off ability. By using different feedback to manipulate task requirements, participants were asked to memorise colours in different precision conditions. We conducted two experiments using a ratio of duration/set size higher than the predicted critical value (Experiment 1) and a ratio lower than the predicted value (Experiment 2).

We anticipated that, at the population level, with a high ratio of duration/set size provided in Experiment 1, we would find that participants in the high VWM capacity group could trade off the precision and number according to task requirements, but participants in the low VWM capacity group could not. However, with a low ratio provided in Experiment 2, neither high VWM capacity participants nor low VWM capacity participants could trade off the precision and number. Moreover, at the individual level, with a high ratio of duration/set size provided in Experiment 1, we would see a relation between trade-off ability and VWM capacity, as for most participants, they would have completed the early phase and entered the late phase of the two-phase model in which allocation could be controlled voluntarily according to task requirements. In contrast, with a low ratio provided in Experiment 2, we would not see such a relation, as for most participants, the processing would still be in the early phase of the model in which VWM resource could be allocated only by an involuntary manner.

## Experiment 1

Based on the study by Ye, *et al*.^21^, when 500 ms exposure duration was used for four memory items, participants were able to trade off the VWM precision and number, we adopted this ratio of 125 ms/item in our Experiment 1.

### Methods

#### Participants

A total of 26 undergraduate students (21.19 ± 1.02 years old, age range 18–22 years; 21 females) were recruited from the participant pool at the Minnan Normal University of China. They reported normal or corrected-to-normal vision and no history of neurological problems. Participants gave informed consent before participating in the experiment. All procedures were approved by the Ethics Committee of the Liaoning Normal University, China, to collect data outside the campus (in Minnan Normal University), and conducted in accordance with the Declaration of Helsinki (2008).

#### Materials

In the change detection task for VWM capacity measurement described below, colour squares (each 0.65° × 0.65°) in the memory array displayed within a 9.8° × 7.3° region around a central fixation cross, with a constraint that the distance between two colours was at least 3.5° (centre-to-centre). The colours were randomly selected from 360 possible colours (1–360, in 1 colour step) in the colour recall task, with colour separated by at least 50 colour steps.

In the colour recall task described below, for the memory array, the colour stimuli were four coloured squares (each 0.9° × 0.9°) presented against a grey background. With a fixation cross in the centre, the colour stimuli were presented at the four corners of an imaginary square with an eccentricity of 3.0°. Then, a set palette of 360 colours in a colour wheel (outer diameter, 7.0°; inner diameter, 5.5°), adopted in a prior study by our group^29^, was used for the recall array. The stimuli showed in the memory array were randomly chosen from the colour palette with a minimum distance of 30° in colour space between stimuli in the array of four colours. The whole experiment was conducted in a dark room, with a 19 inch LCD screen (1280 × 768 pixel) for presenting the stimuli, and the distance between the participant and the screen was approximately 60 cm.

#### Procedure

**Figure 1:**
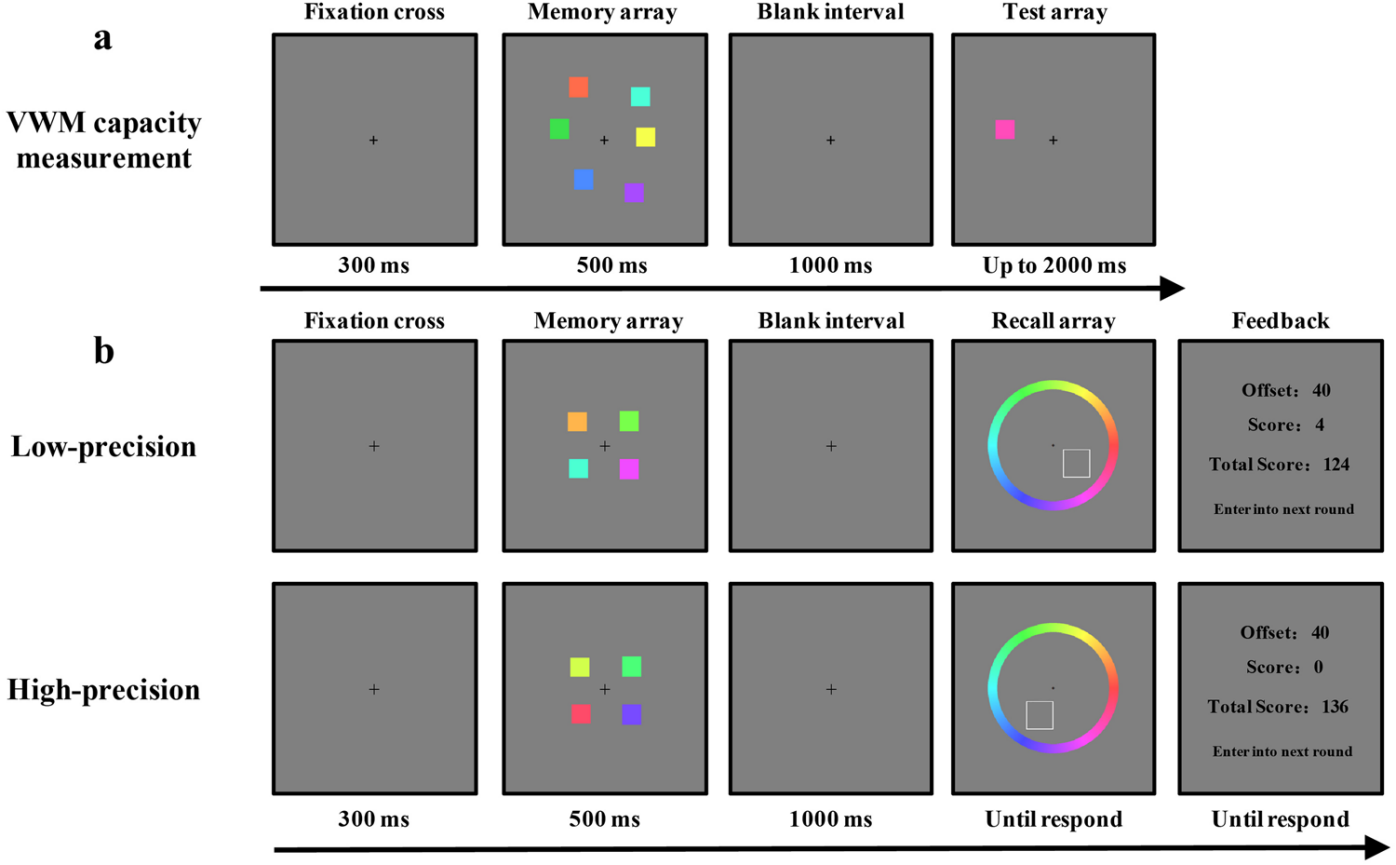
Experimental procedures of Experiment 1. (**a**) Experimental procedure used for the VWM capacity measurement. (**b**) Experimental procedure used in the colour recall task.

There were two sessions in the experiment, the VWM capacity measurement session and the colour recall task session. The participants first completed a change detection task to measure their VWM capacities. In this session (VWM capacity measurement), each trial began with a central fixation cross that appeared on the screen for 200 ms. A memory array of six colours was then presented for 500 ms. After a subsequent 1000 ms retention period featuring a blank screen, a test array that lasted for 2500 ms was presented (Fig. 1a). A test coloured square was presented at the location of one of the sample squares in the memory array. Participants were asked to indicate whether the colour of test square was identical in colour to the corresponding item in the memory array, or whether the colour in the corresponding location changed between the memory and test array. Response accuracy was emphasised rather than response speed. The accuracy was recorded for analysis.

After the completion of the VWM capacity measurement, participants had a rest of at least five minutes before beginning the second session. In this session, participants needed to complete a colour recall task with two precision conditions (Fig. 1b). Each trial started with a fixation cross in the centre of the screen, followed by a 500 ms memory of four colour squares. After a 1000 ms retention period, a recall array was presented. The recall array consisted of an outlined square (1.0° × 1.0°) at the location of one of the sample squares in the memory array in addition to a colour wheel. Participants were asked to report the colour of the remembered item at the location of the white square outline by clicking a mouse on the corresponding location on the colour wheel. Participants were given unlimited time to respond. Then, feedback that contained three values was provided to participants, the “Offset” value, “Score” value and “Total Score” value. The “Offset” value represented the absolute value of the difference between their reported colour value and the actual colour value of the test sample square. The “Score” value represented the scores earned by participants in this trial. The “Total Score” value represented the scores they had accumulated in this condition of the experiment. Participants were encouraged to get higher scores in the colour recall task. The precision (low- and high-precision) conditions were manipulated by varying the rule of the reward scheme. In the low-precision condition, participants were asked to memorise colours in a low-precision way, and earned four scores if their offset was less than 60° in colour space but earned nothing for the offset between 60° and 100° in colour space. In order to encourage participants to memorise as many items as possible in the low-precision condition, participants were penalised two scores for wild guesses (offsets more than 100° in colour space). In the high-precision condition, participants were asked to memorise colours in a high-precision way, and earned six scores if their offset was less than 20° in colour space but earned nothing otherwise. At the beginning of each condition, participants had 100 points as the original score. They were fully informed of the rules of the reward scheme in each condition before the task.

There were 100 trials in the VWM capacity measurement session. In the colour recall task session, the order of the precision conditions was counterbalanced across participants. Each precision condition included 280 trials. There were seven 30-second breaks within-blocks and a 2-min break between the low- and high-precision blocks. Before each block, participants needed to finish at least 24 practice trials to understand the rules. The entire experiment lasted for approximately one hour.

#### Data analysis

For each participant in the VWM capacity measurement session, we measured the VWM capacity by using Cowan’s K formula: K = N × (H − F), where K represents the VWM capacity, N represents the number of displayed colours, H represents the hit rate, and F represents the false alarm rate^30^. Similar to previous VWM studies^13,14,18,31^, the median split was used to divide participants into different VWM capacity groups.

In the colour recall task session, it may have taken a certain amount of time for forming an appropriate strategy based on the task feedback in different conditions. The same method was used here as that used in Ye, *et al*.’s^21^ study, where the first 80 trials in each block were not included because participants need to take a certain amount of time for forming an appropriate strategy based on the task feedback in different conditions. Thus, the results were only calculated based on the last 200 trials. The offset in each trial was calculated by subtracting the recalled colour (the value in colour space) from the target colour.

The data were fitted with the standard mixture model to independently measure the SD (circular standard deviation of a normal distribution, inversely related to the precision of the representations) and G (guess rate) of the representations stored in VWM^32^. The mixture model allowed us to estimate the correct memory rate (P) that the target item was correctly recalled from VWM as 1 − G_._ Data analysis was performed using the MemToolbox^33^. The standard mixture model was fitted to individual data in different precision conditions, respectively. Since the precision is inversely related to the variance, we used the SD (inversely related to the precision) as the memory precision index and used the P (i.e., 1 − G) as the memory number index. Besides, we also used the swap model^34^ to fit the data, as the outcomes from statistical tests of mixture model parameters and swap model parameters are substantially identical (Supplemental Materials).

For the individual level analysis, we defined the voluntary trade-off magnitude in the VWM precision index (SDT) as

$${\rm{SDT}}=\,\frac{\mathrm{SD}(low)-\mathrm{SD}(high)}{{\rm{SD}}(low)}$$

and, defined the voluntary trade-off magnitude in the VWM number index (PT) as

$${\rm{PT}}=\,\frac{P(low)-{\rm{P}}(high)}{P(low)}$$

Then, we merged these two indexes (assuming equal weighting) and calculated a general voluntary trade-off index (GT), which was defined as

$${\rm{GT}}=\,\frac{\mathrm{SD}(low)-{\rm{SD}}(high)}{{\rm{SD}}(low)}+\frac{P(low)-{\rm{P}}(high)}{P(low)}$$

Where SD(*low*) and SD(*high*) are the precision index in the low- and high-precision conditions, and P(*low*) and P(*high*) are the correct memory rate in the low- and high-precision conditions. If the participants allocated resources flexibly in the high-precision conditions, resulting in remembering fewer items, this would make P(*low*) > P(*high*), which would obtain a positive PT. However, the items remembered would be maintained more precisely, meaning SD(*low*) > SD(*high*) (because the SD value is inversely correlated to precision, a larger SD value means a lower precision), which would obtain a positive SDT. By dividing the numerators by the low-precision value, the equations of SDT and PT could minimise the inter-participant variance of the precision and number indexes and indicate the change degree of the indexes in high-precision condition to that in low-precision condition. GT value, a combined measure that contains both precision and number index, is thus the extent to which participant did trade-off according to the task requirements.

For the population level analysis, a significance level of p < 0.05 was used for all variables and was introduced as the criterion for the post hoc analysis in the repeated measures ANOVAs. Paired two-tailed t-tests were conducted for comparison in both capacity groups. Also, Bayes factor analysis was conducted to avoid that the null results were observed by chance^35^. We used the JASP 0.7 statistics package to calculate the Bayes factors^36^. The suggested default priors from JASP have been chosen for the Bayesian analysis. The Bayes factor (*BF*_*10*_, an odds ratio for the alternative/null hypotheses, values < 1 favour the null hypothesis and values > 1 favour the alternative hypothesis) provides a continuous measure of how much more likely the data are under the alternative hypothesis compared with the null hypothesis. In our study, the null hypothesis was that there was no difference between two precision conditions, while the alternative hypothesis was that there was a difference between them. For instance, a *BF*_*10*_ of 0.5 represents that the data are 2 times likely under the null hypothesis than the alternative hypothesis, and a *BF*_*10*_ of 2 represents that the alternative hypothesis is 2 times more likely than the null hypothesis.

For the individual level analysis, as we mentioned above, we expected that if VWM capacity affects the trade-off ability, there should be a positive correlation between VWM capacity (K) and trade-off ability (SDT, PT and GT). Participants with higher VWM capacity should have better trade-off ability. On the contrary, if VWM capacity does not affect the trade-off ability, there should be no correlation between VWM capacity and trade-off ability. Therefore, we used the one-tailed tests in the correlation analysis based on our expectation.

### Results and Discussion

#### Results of different capacity groups (population level)

Participants were divided into different groups by the median split of their VWM capacity. A high VWM capacity group (K = 3.42 ± 0.56) and a low VWM capacity group (K = 1.89 ± 0.58), resulting in 13 participants in each group.

**Figure 2:**
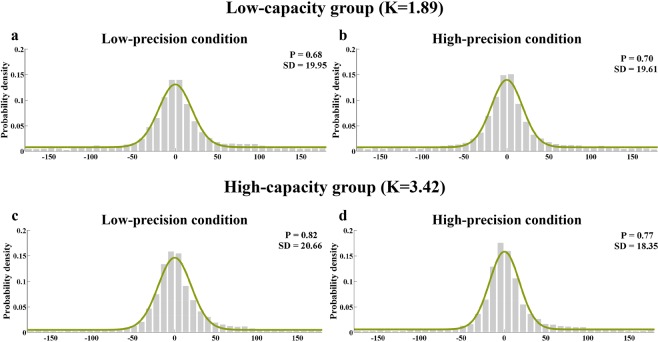
Model-fit results of Experiment 1. The graphs show the model-fit results of the low-capacity group in the top row and the high-capacity group in the bottom row. The results show probability density functions for the offset of responses in the (**a**) low-precision condition in the low-capacity group, (**b**) high-precision condition in the low-capacity group, (**c**) low-precision condition in the high-capacity group, and (**d**) high-precision condition in the high-capacity group. The mean memory number index (P) and precision index (SD) are also shown for each condition.

**Figure 3:**
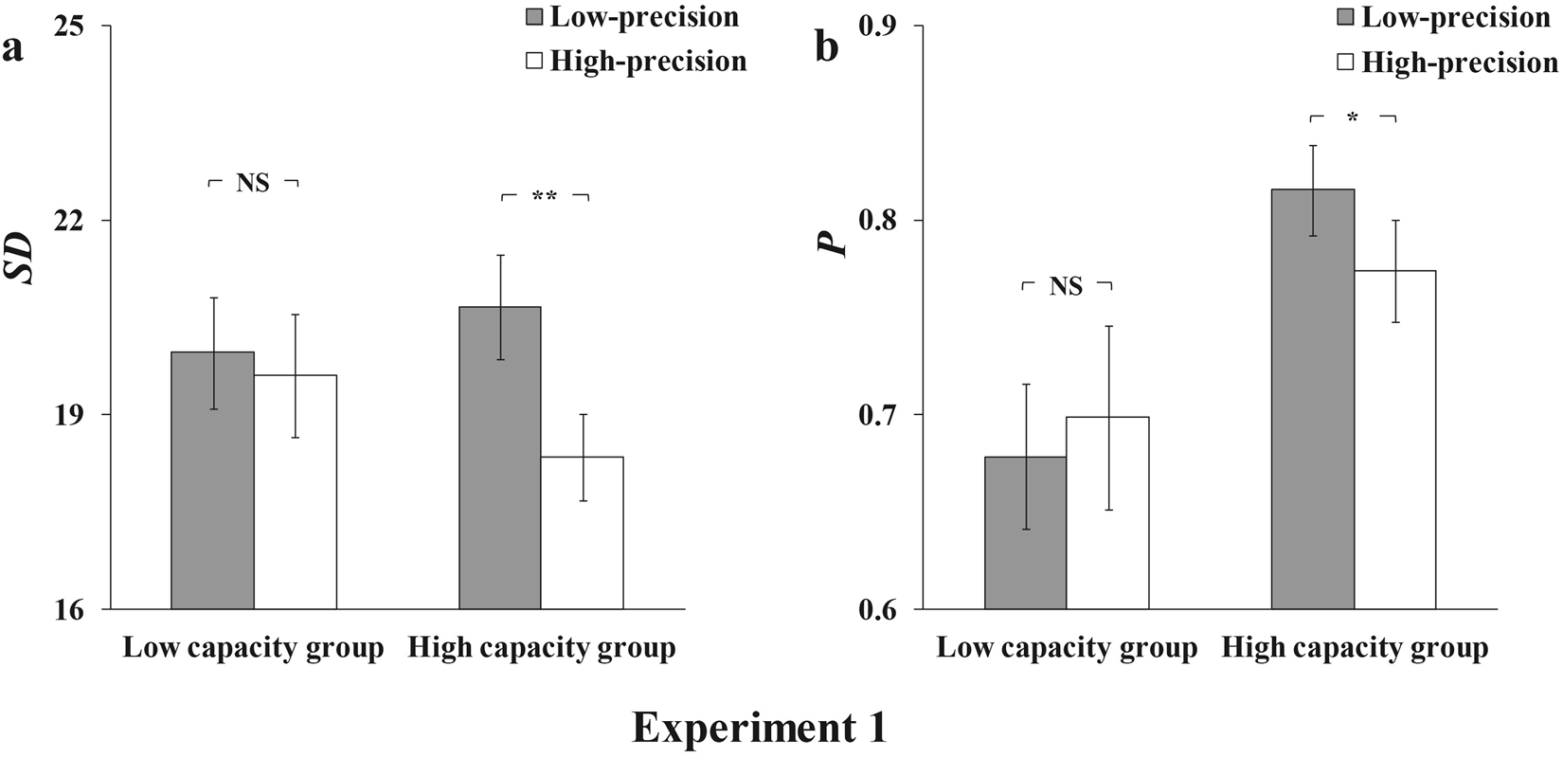
Results for the low- and high-capacity groups in Experiment 1. The graph shows both low-capacity and high-capacity groups’ results, with (**a**) the memory precision index (SD) and (**b**) memory number index (P) presented separately for the low-precision and high-precision conditions. Error bars are standard error of the mean. NS = non-significant; *p < 0.05; **p < 0.01.

We fitted the mixture model to the aggregate data (Fig. 2) and averaged parameters of the mixture model for individual fits. The results of Experiment 1 are found in Fig. 3.

A two-way ANOVA with precision condition (low-precision vs high-precision) and VWM capacity (low VWM capacity vs high VWM capacity) was conducted on the memory precision (SD) and number index (P), respectively. For the precision index (SD), the main effect of precision condition was significant, *F*(1,24) = 13.015, *p* < 0.01, *η*^2^ = 0.352, but the main effect of VWM capacity was non-significant, *F*(1,24) = 0.062, *p* = 0.805, *η*^2^ = 0.003. The interaction between the precision condition and VWM capacity was significant, *F*(1,24) = 7.097, *p* < 0.05, *η*^2^ = 0.228. For the memory number index (P), the main effect of VWM capacity was significant, *F*(1,24) = 4.959, *p* < 0.05, *η*^2^ = 0.171, but the main effect of precision condition was non-significant, *F*(1,24) = 0.812, *p* = 0.377, *η*^2^ = 0.033. The interaction between the precision condition and VWM capacity was significant, *F*(1,24) = 6.625, *p* < 0.05, *η*^2^ = 0.216.

Follow-up pairwise comparisons showed that, for the high VWM capacity group the VWM precision was higher for the high-precision condition compared to the low-precision condition, *t*(12) = 4.102, *p* < 0.001, *Cohen’s d* = 0.86, *BF*_*10*_ = 28.57 for SD. Besides, the memory number in the high-precision condition was less than that in the low-precision condition, *t*(12) = 2.621, *p* < 0.05, *Cohen’s d = *0.47, *BF*_*10*_ = 3.03 for P. In contrast, for the low-capacity group, there was neither VWM precision difference nor memory number difference between high-precision and low-precision conditions, *t*(12) = 0.732, *p* = 0.478, *Cohen’s d* = 0.11, *BF*_*10*_ = 0.35 for SD; *t*(12) = 1.117, *p* = 0.286, *Cohen’s d* = 0.13, *BF*_*10*_ = 0.47 for P.

In line with our expectation, these results support the hypothesis that VWM capacity affects the trade-off ability. The results suggest that for the high-capacity group, participants memorise fewer colours but with higher precision in the high-precision condition compared to the low-precision condition. However, for the low-capacity group, there was no significant difference in the precision or number between two precision conditions.

#### Correlation results (individual level)

**Figure 4:**
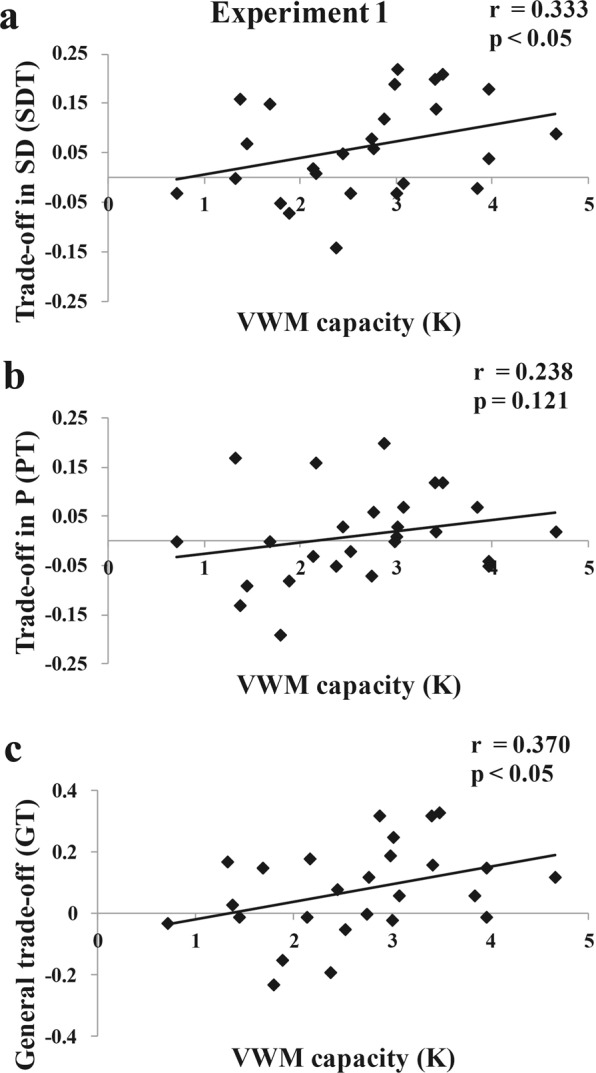
Correlation results of Experiment 1. (**a**) Correlation between VWM capacity(K) and trade-off magnitude in precision (SDT), (**b**) Correlation between VWM capacity(K) and trade-off magnitude in number (PT), and (**c**) Correlation between VWM capacity(K) and general trade-off index(GT).

We measured the relationship between the K (VWM capacity) and SDT (trade-off magnitude in VWM precision) value, K and PT (trade-off magnitude in VWM number) value, K and GT (general trade-off magnitude) value. The SDT, PT, GT values were plotted as a function of each’s VWM capacity in Fig. 4a–c.

The K and SDT were positively correlated (*r* = 0.333, *p* < 0.05, one-tailed): low VWM capacity participants showed low trade-off magnitude in VWM precision, and high VWM capacity participants produced much higher trade-off magnitude in VWM precision. There was a small positive correlation trend between the K and PT, but it was not statistically significant (*r* = 0.238, *p* = 0.121, one-tailed). More importantly, the VWM capacity and GT were positively correlated (*r* = 0.370, *p* < 0.05, one-tailed): low VWM capacity participants showed low general trade-off magnitude, and high VWM capacity participants produced much higher general trade-off magnitude.

In the results of the population level analysis, we found that there was an impact of VWM capacity on the trade-off ability. Participants in the high VWM capacity group were able to trade off the VWM precision and number voluntarily, but those in the low VWM capacity group had difficulty in a flexible trade-off. More importantly, in the individual level analysis, the results of Experiment 1 showed that the VWM capacity of the individual was positively correlated with the trade-off ability when participants have enough consolidation time to trade off the VWM precision and number. The pattern of results found here seems to be consistent with the results found by Vogel, *et al*.^13^, suggesting that low VWM capacity individuals failed to filter out the distractors. In other words, it might take extra effort to consolidate the memory selectively. Low VWM capacity individuals might forgo the extra selective processing because it is self-defeating or uncomfortably effortful for them^17^. The extra processing may also encourage low VWM capacity participants to skip the voluntary trade-off process in our study.

According to the two-phase model and our hypothesis, the trade-off pattern of individuals may be different with different critical values of duration/set size. Consequently, we wanted to verify this in Experiment 2 with a smaller ratio of duration/set size.

## Experiment 2

The purpose of Experiment 2 was to verify whether the smaller ratio of duration/set size would cause the failure of the voluntary trade-off for most participants (and consequently lack of relation between voluntary trade-off magnitude and VWM capacity) as suggested by the two-phase model. We used the same procedure as Experiment 1 but reduced the exposure duration from 500 ms to 200 ms (the set size remained as four), generating a ratio of duration/set size of 50 ms/item. It is expected that participants could not voluntarily trade off for the small ratio of duration/set size as they need more time to enter the late allocation phase.

### Methods

#### Participants

Twenty-six new undergraduate students (20.99 ± 1.07 years old, age range 19–24 years, 23 females) were recruited from the participant pool at the Minnan Normal University. All procedures were approved by the Ethics Committee of the Liaoning Normal University, China, to collect data outside the campus (in Minnan Normal University), and conducted in accordance with the Declaration of Helsinki (2008).

#### Procedure and data analysis

Except that the exposure duration of the memory array in the colour recall task decreased from 500 ms to 200 ms, the procedure and data analysis of Experiment 2 was identical with that of Experiment 1.

### Results and Discussion

#### Results of different VWM capacity groups (population level)

As in Experiment 1, we divided 26 new participants based on their VWM capacity estimated in Experiment 2 into two different groups, a high VWM capacity (K = 3.24 ± 0.59) and a low-capacity group (K = 1.81 ± 0.48), resulting in 13 participants in each group respectively.

**Figure 5:**
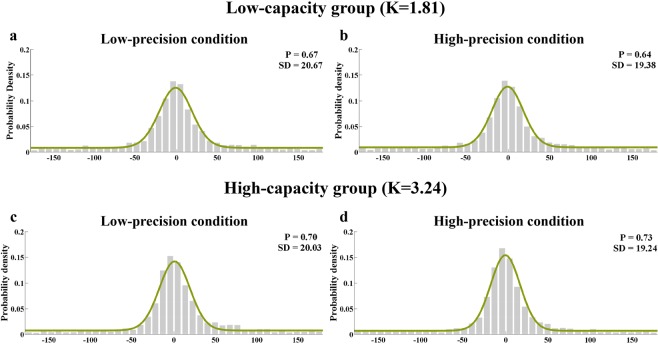
Model-fit results of Experiment 2. The graphs show the model-fit results of the low-capacity group in the top row and the high-capacity group in the bottom row. The results show probability density functions for the offset of responses in the (**a**) low-precision condition in the low-capacity group, (**b**) high-precision condition in the low-capacity group, (**c**) low-precision condition in the high-capacity group, and (**d**) high-precision condition in the high-capacity group. The mean memory number index (P) and precision index (SD) are also shown for each condition.

**Figure 6:**
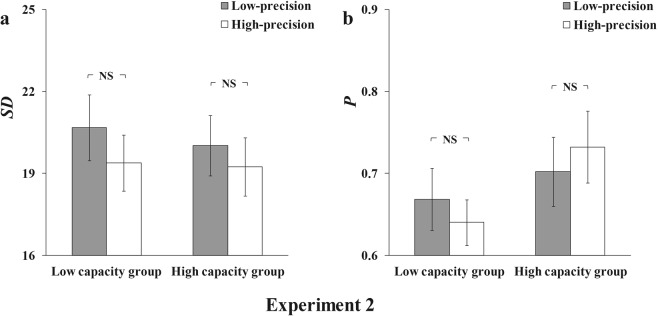
Results for the low- and high-capacity groups in Experiment 2. The graph shows both low-capacity and high-capacity groups’ results, with (**a**) the memory precision index (SD) and (**b**) memory number index (P) presented separately for the low-precision and high-precision conditions. Error bars are standard error of the mean. NS = non-significant.

Again, the mixture model was fitted to the aggregate data (Fig. 5) and averaged parameters of the mixture model for individual fits. The results were shown in Fig. 6.

A two-way ANOVA with precision condition (low-precision vs high-precision) and VWM capacity (low VWM capacity vs high VWM capacity) was conducted on the memory precision (SD) and number index (P), respectively. For the memory precision index (SD), the main effects of precision condition, *F*(1,24) = 0.883, *p* = 0.357, *η*^2^ = 0.035, and VWM capacity, *F*(1,24) = 0.129, *p* = 0.722, *η*^2^ = 0.005, were non-significant. Also, there was no significant interaction effect between the precision condition and VWM capacity, *F*(1,24) = 0.053, *p* = 0.820, *η*^2^ = 0.002. For the memory number index (P), similar to the result pattern of memory precision index, no significant main effect was found neither for precision condition, *F*(1,24) = 0.002, *p* = 0.967, *η*^2^ = 0.000, nor for VWM capacity, *F*(1,24) = 1.604, *p* = 0.218, *η*^2^ = 0.063. Also, the interaction effect between the precision condition and VWM capacity was non-significant, *F*(1,24) = 1.827, *p* = 0.189, *η*^2^ = 0.071.

The results suggest that for both high and low VWM capacity group, participants maintained the same number of VWM representations with the same precision in different precision conditions. In line with our expectation, that is, with a small ratio of duration/set size, both low- and high-capacity groups did not show a trade-off ability as it appeared in Experiment 1. In other words, with a smaller ratio, most people can only allocate VWM resource in a stimulus-drive (involuntary) manner regardless of their VWM capacity.

In addition, a mixed-factor ANOVA was conducted respectively for on the memory precision (SD) and number index (P), with the precision condition (low-precision vs high-precision) as a within-subject factor, and the VWM capacity (low VWM capacity vs high VWM capacity) and experiment (Experiment 1 vs Experiment 2) as between-subject factors For the memory precision index (SD), a significant main effect of precision condition was found, *F*(1,48) = 4.135, *p* < 0.05, *η*^2^ = 0.079, but no other significant main effect nor interaction was found (all *ps* > 0.294). For the memory number index (P), the main effect of VWM capacity was significant, *F*(1,48) = 6.025, *p* < 0.05, *η*^2^ = 0.112, and there was a significant interaction effect across precision condition, VWM capacity and experiment, *F*(1,48) = 5.909, *p* < 0.05, *η*^2^ = 0.110, but neither other significant main effects nor interaction was found (all *ps* > 0.112).

The results of ANOVA across experiments showed that there was a significant interaction between two precision conditions, VWM capacity and experiment for the number index, but no significant interaction for the precision index. However, in Experiment 1 there was a significant positive correlation between VWM capacity and trade-off magnitude in precision, but no significant positive correlation between VWM capacity and trade-off magnitude in number. These different result patterns may be due to the different variability of number index and precision index, the difference of number index was more evident in the population level analysis, on the contrary, the difference of precision was more evident in the individual level analysis. Therefore, we respectively reported the results of the population level analysis and individual level analysis. It would help us to have a more comprehensive understanding of the relationship between VWM capacity and voluntary trade-off ability.

#### Correlation results (individual level)

**Figure 7:**
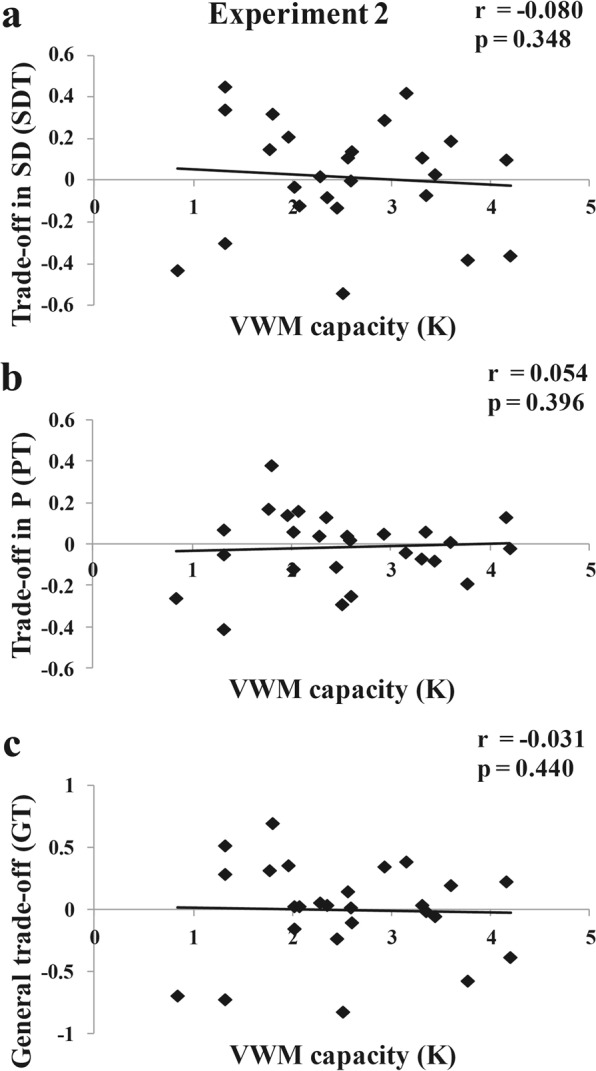
Correlation results of Experiment 2, (**a**) Correlation between VWM capacity (K) and trade-off magnitude in precision (SDT), (**b**) Correlation between VWM capacity (K) and trade-off magnitude in number (PT), and (**c**) Correlation between VWM capacity (K) and general trade-off index (GT).

Again, we measured the relationship between the K (VWM capacity) and SDT (trade-off magnitude in VWM precision) value, K and PT (trade-off magnitude in VWM number) value, K and GT (general trade-off magnitude) value. The SDT, PT, GT values were plotted as a function of each’s VWM capacity in Fig. 7a–c. The results showed no correlations between the K and SDT (*r* = −0.080, *p* = 0.348, one-tailed), K and PT (*r* = 0.054, *p* = 0.396, one-tailed), K and GT (*r* = −0.031, *p* = 0.440, one-tailed).

The results of both population level and individual level analyse in Experiment 2 suggest that regardless of the VWM capacity, participants could not voluntarily trade off the VWM precision and number under a small ratio of duration/set size condition. The present evidence supports that the voluntary trade-off failed in Experiment 1 of Ye, *et al*.’s^21^ study only because of insufficient exposure duration. Thus, the result further verifies the two-phase model.

## General Discussion

Experiment 1 showed, with a high ratio of duration/set size, that high VWM capacity participants were able to adjust memory precision flexibly according to task requirements, while low VWM capacity participants failed. More importantly, the VWM capacity of the individual was positively correlated with the ability of voluntary trade-off when most participants, for a given set size, have enough consolidation time to trade off the VWM precision and number. Following the previous demonstration that the individual’s VWM capacity could affect the ability of attention control to filter distractors^11,13^, our work showed that VWM capacity is also related to attention control in VWM task in allowing a voluntary trade-off between VWM precision and number, that is, the ability of voluntary VWM resource allocation. Although many previous studies have explored whether individuals can voluntarily trade off the VWM precision and number^19–21,23^, based on our knowledge, the current study provides the first evidence that there is a correlation between the ability of voluntary trade-off and the VWM capacity when the value of duration/set size is large enough (larger than the critical value of 100 ms/item). For participants with low VWM capacity who showed a weaker trade-off ability, the resource allocation is mainly in the form of a stimulus-driven manner, while for participants with high VWM capacity, they can take extra effort to allocate resource to trade off the precision and number voluntarily. In contrast, in Experiment 2, when the ratio of duration/set size was only 50 ms/item (well below the critical value of 100 ms/item), the results showed that regardless of the VWM capacity, most participants could not voluntarily trade off the VWM precision and number. In this case, there was no relation between VWM capacity and voluntary trade-off ability.

In the present study, we not only reported the results of the population level analysis on the memory precision and number index respectively as previous studies^21,23,25,26,28^, but also the results of the individual level analysis by merging precision and number index. The population level analysis for the precision index did not reach significance in a between-experiment analysis (i.e., the three-way interaction of precision condition × capacity group × experiment on the precision index). This may reflect some potential problems of the population level analysis, such as the relying solely on a median split to divide capacity groups for data analysis (while useful for illustrative purposes) might be problematic from a statistical standpoint. Thus, the continuous measure of performance (e.g., individual level analysis) may be a more appropriate approach here. The discussion below is mainly based on the correlation results within the separate experiments.

### Accounting for our results and others published with the two-phase model

In general, the results from the current study are consistent with the two-phase model proposed by Ye, *et al*.^21^, suggesting that for a given set size, when the consolidation time is extremely short, regardless of VWM capacity, individuals cannot allocate resources voluntarily. However, when the consolidation time is long enough, individuals can allocate resources voluntarily, and the ability to allocate resources is highly related to their VWM capacity.

In previous studies about the voluntary trade-off between VWM precision and number, many studies supported the voluntary trade-off used the orientation as the stimulus^19–26^, but most of the studies using colour materials failed to find evidence that participants could trade off voluntarily^22,23,25^. Therefore, one might think that the stimulus property (orientation vs colour) might be the determining factor for the patterns of the results. After all, orientation is a boundary feature while the main property of a colour stimulus is its surface feature^37^. The consolidation mechanisms for the orientation and colour materials could be different. For example, a series of studies have shown that the memory consolidation bandwidth is different between colour and orientation feature^38–43^. Thus, there is a possibility that individuals could show a lack of voluntary trade-off ability for colour materials. However, the results of our Experiment 1 showed that there was a voluntary trade-off for colour materials (also see Fougnie, *et al*.^26^). These results suggest that, regardless of the stimulus property, the individuals could voluntarily trade off the VWM precision and number with long exposure duration. Moreover, it should be noted that, as it showed in our Experiment 2, with a small ratio of duration/set size, the individuals could not trade off voluntarily in the condition. Therefore, although the two-phase model was proposed in the study using orientation materials as stimulus^21^, the combined results of our Experiment 1 and 2 suggest that the model is also suitable for colour materials.

It is important to point out that, as mentioned in the introduction, there were still two pieces of contrary evidence (2/25 experiments) which did not correspond to the predictions of the two-phase model. The first contrary evidence was the result of a supplementary experiment in Fougnie, *et al*.’s^26^ study. Although the two-phase model could well predict the results of their Experiment 1 and 2 (which could trade off the VWM precision and number when participants memorize five colours at 1200 ms exposure duration), they conducted a quick supplementary experiment and found that participants could also trade off the VWM precision and number when they needed to memorize five colours at 200 ms. Thus, their study suggested that participants could trade off VWM precision and number without being affected by the length of exposure duration. The second contrary evidence was Experiment 3 in Ramaty and Luria’s^28^ study. Ramaty and Luria^28^ conducted a follow-up study of Fougnie, *et al*.^26^’s study, but they found different results from Fougnie, *et al*.’s^26^ study. In Ramaty and Luria’s^28^ study, they firstly repeated that participants could trade off the VWM precision and number when they need to memorise five items at 1200 ms exposure duration (Experiment 1). Then they found that this trade-off disappeared when the exposure duration decreases from 1200 ms to 300 ms (Experiment 2). Although the results mentioned above (the first two experiments in Ramaty and Luria’s^28^ study) could be predicted by the two-phase model, Experiment 3 in Ramaty and Luria’s^28^ study showed that the trade-off disappeared when participants need to memorise five items at 1200 ms exposure duration with an articulatory suppression. Thus, they suggested that an effective trade-off between VWM precision and number was due to verbal encoding instead of VWM processing, and the verbal encoding was mediated by long encoding durations. Unlike the present study, both of these studies^26,28^ asked participants to conduct a standard task (for the high-precision condition) and a get-them-all task (for the low-precision condition) to induce a trade-off. In the standard task, participants were asked to report only one colour out of five colours that appeared in the memory array, but in the get-them-all task, participants were asked to report all five colours. Compared with the standard task, participants had more motivation to maintain all five colours simultaneously in the get-them-all task. Because memorising five colours are beyond the average of individual VWM capacity, participants were not able to merely use the VWM encoding to complete the get-them-all task, which leads to the participants may use verbal encoding as a strategy to improve the performance, thus producing a stronger trade-off effect. However, the verbal encoding hypothesis could not explain our current results because of the following reasons. Firstly, in our study, the response requirements were the same in low- and high-precision conditions. Even if the verbal encoding can assist memory, participants should use verbal coding to help to memorise under both conditions. Thus, the effect of verbal encoding should be counteracted by calculating trade-off indexes. Secondly, if the trade-off was caused by using verbal encoding to memorise items strategically, we should be able to observe that low VWM capacity participants could also trade off effectively as high VWM capacity participants in our Experiment 1. Thirdly, verbal encoding requires a long retention interval or exposure duration. For example, in Experiment 4 of Souza and Skora’s^44^ study, they found that the articulatory suppression impaired the performance of the VWM task when the four colours were present at a 250 ms exposure duration followed by a 3000 ms retention interval. However, the articulatory suppression did not affect the performance of the VWM task when the four colours were present at a 250 ms exposure duration followed by a 1000 ms retention interval. In Vogel, *et al*.’s^45^ study, they found that there was no difference in the memory performance between the 100 ms exposure duration and 500 ms exposure duration when the participants needed to memorise four colours. Moreover, the VWM performance did not change because of the additional verbal working memory load. This evidence suggests that verbal encoding has a limited impact on VWM performance in our study, which used 500 ms or less exposure duration and 1000 ms retention interval. Therefore, the trade-off in the present study is mainly caused by the reallocation of VWM resources, instead of verbal encoding.

### Critical value of duration/set size may be related to memory consolidation

Both the current results and results found in Ye, *et al*.’s^21^ study demonstrated the importance of the critical value of duration/set size as a factor in determining whether voluntary trade-off would occur. The current results showed that when a ratio of 125 ms/item was adopted for presenting stimuli, participants with high VWM capacity could flexibly adjust the trade-off according to task requirements. However, participants could not voluntarily adjust the trade-off regardless of the VWM capacity when the ratio of 50 ms/item was adopted. According to the two-phase model, whether individuals can start the late resource allocation phase is mainly depends on their early consolidation processing. The consolidation process, the initial formation of VWM representations, has been studied for decades^38,46–49^. Vogel, *et al*.’s^48^ study showed that the VWM consolidation rate of the colour stimulus was about 50 ms/item^48^. A recent study by our group found similar results that participants spent an average of 60 ms to consolidate a colour stimulus into their VWM^38^. Thus, these results imply that the rate of duration/set size allowing individuals to trade off voluntarily is probably related to the time spent on VWM consolidation of the stimuli, and the voluntary trade-off between quality and quantity only occur when the ratio for presenting stimuli is much larger than the rate of consolidation.

### Trade-off issue in VWM models

It is important to point out that the trade-off of precision and number can take place in two forms. Participants can opt to increase memory precision by sacrificing the number of items remembered. A more interesting question would be whether participants can increase the VWM number by sacrificing precision. It is still under debate if such an increase in VWM number can go beyond an upper limit of normal memory capacity^23,26^.

For the nature of VWM resource, there are two classes of models: “slot” model and “resource” model. The classical slot model proposes that the number of available resources (considered as “slots”) in VWM is limited. The slots can only be used to keep a limited set of discrete items. It means that when all those slots are fully occupied in VWM, no additional items can be further stored into these slots, and the memory precision could not be affected by the number of memory items^30,45,50^. However, a more recent revision of such a model, called slot + average model, states that when the number of stored items did not reach the maximal number of slots, a single item could be stored in multiple slots, leading to an increase in precision of that item^32^. In contrast, flexible resource model suggests a flexible allocation of limited cognitive resources. Allocating more resources to an item will allow greater precision. The trade-off between precision and number can be in two directions: an increase in precision on a small number of items or increase in memory number by sacrificing precision. More importantly, the resource models do not impose an upper limit on memory number^34,51–54^. There have been studies supporting each of the two classes of models mentioned above. The current study was not designed to provide direct support for either model. Instead, we offered a novel perspective suggesting that, for a given set size, the stimulus duration is the determining factor for the voluntary trade-off. The current study utilised the set size of four, which is likely for most participants within their VWM capacity. Future work may introduce tasks requiring participants to memorise a large number of the items above estimated average memory capacity to seek an answer for the question whether there is a limit in storing more items by scarifying precision.

### Other contributions to future research

The present study showed that when participants were able to trade off the VWM precision and number voluntarily, the individual’s VWM capacity is positively related to the voluntary trade-off ability for VWM precision and number. This result is consistent with the results found in VWM task involving distractors, which suggests that there is a positive correlation between VWM capacity and attention filtering ability^11,13^. However, the attention mechanism invoked in the studies about attention filtering may not be entirely the same as the attention that is invoked in the present study. The mental process involved in ignoring distractors might be primarily contributed by selective attention, which has been widely studied in the interaction between VWM and attention^55–58^. This kind of attention can be classified as external attention^58^. In addition to this, another kind of attention called internal attention also affects VWM performance, which is used by participants in the present study to reallocate resource to achieve a voluntary trade-off between VWM quality and quantity. The impact of internal attention on VWM processing has been generating considerable interest. For example, recent studies suggested that more internal attention resources enable individuals to allocate VWM resources more effectively, thereby improving VWM performance^59^. In addition, individuals can use internal attention to flexibly allocate VWM resources to even a particular dimension of representations^29,60^. Thus, the present study provides an important supplement to the research on the relation between attention and VWM.

It is worth noting that although there have been many studies on trade-off using a mixture model (or other models) fitting^21,23,25,26,28^, according to our knowledge, all of them analysed the precision and number index respectively. In the present study, we used GT as a measure to merge the precision and number index to quantify the magnitude of voluntary trade-off. We did not use this paper to argue that the equation of GT is an ideal solution to the trade-off issue in the field, but we believe that future studies on trade-off can further improve the equation of GT to take into account the change of both precision and number. For example, future research could further explore the relationship between individual trade-off ability and attentional filtering ability with a method similar to the GT equation. Therefore, the setup of the combined measure in this paper is a new beginning to quantify the individual’s trade-off ability.

## Supplementary information

Data fitting results of swap model

## Data Availability

The datasets generated during and/or analysed during the current study are available from the corresponding author (lq780614@163.com, Qiang Liu) on reasonable request.
