# Supplementary material for: Working memory capacity affects trade-off between quality and quantity only when stimulus exposure duration is sufficient: Evidence for the two-phase model
Source: Sci Rep. 2019 Jun 19;9:8727. doi: 10.1038/s41598-019-44998-3 (PMC6584737; doi:10.1038/s41598-019-44998-3)
Supplement: Supplementary file 1 — Data fitting results of swap model [file 41598_2019_44998_MOESM1_ESM.docx]

Working memory capacity affects trade-off between quality and quantity only when stimulus exposure duration is sufficient: Evidence for the two-phase model

Chaoxiong Ye^a,b^, Hong-Jin Sun^c^, Qianru Xu^b^, Tengfei Liang^d^, Yin Zhang^d^, Qiang Liu^a,d*^

a. Institute of Brain and Psychological Science, Sichuan Normal University, Chengdu, China, 610000

b. Department of Psychology, University of Jyvaskyla, Jyväskylä, Finland, 40014

c. Department of Psychology, Neuroscience and Behaviour, McMaster University, Hamilton, Canada, L8S 4K1

d. Research Center of Brain and Cognitive Neuroscience, Liaoning Normal University, Dalian, China, 116029

***Correspondence to:**

Qiang Liu, PhD.

Professor, Institute of Brain and Psychological Science,

Sichuan Normal University, Chengdu, 610000, China

Telephone: +8613332220573

E-mail: lq780614@163.com

Supplementary Materials

Data fitting results of swap model

In the supplementary materials, the swap model [^1^](#_ENREF_1) was used to fit data by using Memtoolbox [^2^](#_ENREF_2), and then calculated the voluntary trade-off index based on the results of the model fitting. The swap model assumes that in addition to the two types of the standard mixture model (random guess responses and responses based on noisy internal memory) there is third type of responses (non-target memory responses). Thus, there are three main parameters in the results of swap model fitting, memory precision index (SD_s_), guess rate (G_s_) and non-target reported rate (B_s_). Then we estimated the memory number index (P_s_) by calculating the correct response rate (i.e., 1- G_s_ - B_s_).

Based on the memory precision index (SD_s_) and number index (P_s_), the voluntary trade-off magnitude in the VWM number index (P_s_T) was defined as

$$P_{s}T= \frac{P_{s}\left( low \right)-P_{s}(high)}{P_{s}\left( low \right)}$$

and the voluntary trade-off magnitude in the VWM precision index (SD_s_T) was defined as

$$\mathrm{SD}_{s}T= \frac{\mathrm{SD}_{s}\left( low \right)-\mathrm{SD}_{s}(high)}{\mathrm{SD}_{s}(low)}$$

Then, these two indexes were merged and calculated as a general voluntary trade-off index (G_s_T), which was defined as

$$G_{s}T= \frac{P_{s}\left( low \right)-P_{s}(high)}{P_{s}\left( low \right)}+\frac{\mathrm{SD}_{s}\left( low \right)-\mathrm{SD}_{s}(high)}{\mathrm{SD}_{s}(low)}$$

where P_s_(*low*) and P_s_(*high*) represent the correct memory rate in the low- and high-precision conditions, and SD_s_(*low*) and SD_s_(*high*) represent the precision index in the low- and high-precision conditions. G_s_T value represents the magnitude of voluntary trade-off.

Correlation results

**Experiment 1**

The Pearson correlation coefficient was measured between K (VWM capacity) and SD_s_T value (trade-off magnitude in VWM precision), K and P_s_T (trade-off magnitude in VWM number) value, K and G_s_T (general trade-off magnitude) value. The SD_s_T, P_s_T, G_s_T values were plotted as a function of each individual’s VWM capacity in Figure 1a-c. The results showed that there was a significant positive correlation between K and SD_s_T (*r* = 0.362, *p* < 0.05, one-tailed). There was a small positive correlation trend between the K and P_s_T, but it was not statistically significant (*r* = 0.175, *p* = 0.196, one-tailed). More importantly, the K and G_s_T were positively correlated (*r* = 0.348, *p* < 0.05, one-tailed).

**Figure 1. Correlation results based on the swap model fitting in Experiment 1.** *(a) Correlation between VWM capacity(K) and trade-off magnitude in precision (SDsT), (b) Correlation between VWM capacity (K) and trade-off magnitude in number (P_s_T), and (c) Correlation between VWM capacity(K) and general trade-off index (G_s_T).*

Experiment 2

Similar to the analysis used in Experiment 1, in Experiment 2, we measured the relationship between K and SD_s_T value, K and P_s_T value, K and G_s_T value in same way. The SD_s_T, P_s_T and G_s_T values were plotted as a function of each individual’s VWM capacity in Figure 2a-c. The results showed no correlations between the K and SD_s_T (*r* = -0.080, *p* = 0.350, one-tailed), K and P_s_T (*r* = 0.001, *p* = 0.499, one-tailed), K and G_s_T (*r* = -0.054, *p* = 0.396, one-tailed).

**Figure 2. Correlation results based on the swap model fitting in Experiment 2.** *(a) Correlation between VWM capacity(K) and trade-off magnitude in precision (SD_s_T), (b) Correlation between VWM capacity (K) and trade-off magnitude in number (P_s_T), and (c) Correlation between VWM capacity (K) and general trade-off index (G_s_T).*

Results of different VWM capacity groups

Experiment 1

Participants were divided into different groups by the median split of their VWM capacity. A high VWM capacity group (K = 3.42 ± 0.56) and a low VWM capacity group (K = 1.89 ± 0.58), resulting in 13 participants in each group. The parameters of the swap model (P_s_, SD_s_) were calculated for individual fits in Experiment 1. The results were shown in Figure 3.

A two-way ANOVA with precision condition (low-precision vs. high-precision) and VWM capacity (low VWM capacity vs. high VWM capacity) was conducted on the memory precision (SD_s_) and number (P_s_) index, respectively. For the memory precision index (SD_s_), there was a main effect of precision condition [*F*(1,24) = 13.816, *p* < .001, *η^2^* = 0.365], but no significant main effect of VWM capacity [*F*(1,24) = 0.696, *p* = .413, *η^2^* = 0.028]. The interaction between the precision condition and VWM capacity was significant [*F*(1,24) = 8.622, *p* < .01, *η^2^* = 0.264]. For the memory number index (P_s_), there was a main effect of VWM capacity [*F*(1,24) = 4.450, *p* < .05, *η^2^* = 0.156], but no significant main effect of precision condition [*F*(1,24) = 1.806, *p* = .192, *η^2^* = 0.070]. The interaction between the precision condition and VWM capacity was significant [*F*(1,24) = 4.384, *p* < .05, *η^2^* = 0.154].

Follow-up pairwise comparisons showed that, for the high VWM capacity group the VWM precision in the high-precision condition was higher than that in the low-precision condition [*t*(12) = 4.748, *p* < .001, *Cohen's d* = 0.94 for SD_s_]. Besides, the memory number in the high-precision condition was less than that in the low-precision condition [*t*(12) = 2.485, *p* < .05, *Cohen's d* = 0.47 for P_s_]. In contrast, for the low-capacity group, there was neither VWM precision difference nor memory number difference between high-precision and low-precision conditions [*t*(12) = 0.547, *p* = .594, *Cohen's d* = 0.09 for SD_s_; *t*(12) = 0.519, *p* = .613, *Cohen's d* = 0.06 for P_s_].

**Figure 3. Swap model fitting results for the low VWM capacity group and high VWM capacity group in Experiment 1.** *The graph shows the low-capacity (a) and high-capacity (b) groups' results, with the memory number index (P_s_) and memory precision index (SD_s_) separately for the low-precision and high-precision conditions. Error bars are standard error of the mean. NS = non-significant; * = p < 0.05; ** = p < 0.01.*

Experiment 2

As Experiment 1, we divided 26 new participants based on their VWM capacity estimated in Experiment 2 into two different groups, a high VWM capacity (K = 3.24 ± 0.59) and a low-capacity group (K = 1.81 ± 0.48), resulting in 13 participants in each group respectively. Again, the parameters of the swap model (P_s_, SD_s_) were calculated for individual fits in Experiment 2. The results were shown in Figure 4.

A two-way ANOVA with precision condition (low-precision vs. high-precision) and VWM capacity (low VWM capacity vs. high VWM capacity) was conducted on the memory precision (SD_s_) and number (P_s_) index, respectively. For the memory precision index (SD_s_), there were no significant main effects of precision condition [*F*(1,24) = 0.645, *p* = .430, *η^2^* = 0.026] and VWM capacity [*F*(1,24) = 0.014, *p* = .906, *η^2^* = 0.001]. Also, the interaction effect between the precision condition and VWM capacity was non-significant [*F*(1,24) = 0.204, *p* = .656, *η^2^* = 0.008]. For the memory number index (P_s_), similar to the result pattern of memory precision index, there was no significant main effects of precision condition [*F*(1,24) = 0.051, *p* = .823, *η^2^* = 0.002] and VWM capacity [*F*(1,24) = 1.596, *p* = .219, *η^2^* = 0.062]. Also, the interaction effect between the precision condition and VWM capacity was non-significant [*F*(1,24) = 2.263, *p* = .146, *η^2^* = 0.086].

**Figure 4. Swap model fitting results for the low VWM capacity group and high VWM capacity group in Experiment 2.** *The graph shows the low-capacity (a) and high-capacity (b) groups' results, with the memory precision index (P_s_) and memory number index (SD_s_) presented separately for the low-precision and high-precision conditions. Error bars are standard error of the mean. NS = non-significant.*

Reference

1 Bays, P. M., Catalao, R. F. & Husain, M. The precision of visual working memory is set by allocation of a shared resource. *Journal of vision* **9**, 7 1-11, doi:10.1167/9.10.7 (2009).

2 Suchow, J. W., Brady, T. F., Fougnie, D. & Alvarez, G. A. Modeling visual working memory with the MemToolbox. *Journal of vision* **13**, doi:10.1167/13.10.9 (2013).
